# [μ-1,2-Bis(diphenyl­phosphino)methane-κ^2^ *P*:*P*′]bis­{[(*Z*)-*O*-ethyl *N*-(4-nitro­phen­yl)thio­carbamato-κ*S*]gold(I)}

**DOI:** 10.1107/S1600536810016326

**Published:** 2010-05-08

**Authors:** Soo Yei Ho, Edward R. T. Tiekink

**Affiliations:** a Department of Chemistry, National University of Singapore, Singapore 117543; b Department of Chemistry, University of Malaya, 50603 Kuala Lumpur, Malaysia

## Abstract

Each gold atom in the binuclear title compound, [Au_2_(C_9_H_9_N_2_O_3_S)_2_(C_25_H_22_P_2_)], is coordinated within an *S*,*P*-donor set that defines a slightly distorted linear geometry [S—Au—P angles = 172.77 (6) and 173.84 (6)°], with the distortion due in part to a close intra­molecular Au⋯O contact [2.968 (11) and 2.963 (4) Å]. The mol­ecule adopts a U-shaped conformation allowing for the formation of an aurophilic Au⋯Au inter­action [3.2320 (5) Å]. Mol­ecules are consolidated in the crystal structure by C—H⋯π inter­actions. Disorder was noted for one of the eth­oxy groups with two orientations being resolved in a 0.679 (16):0.321 (16) ratio.

## Related literature

For the structural systematics and luminescence properties of phosphinegold(I) carbonimidothio­ates, see: Ho *et al.* (2006 ▶); Ho & Tiekink (2007 ▶); Kuan *et al.* (2008 ▶). For the synthesis, see: Hall *et al.* (1993 ▶).
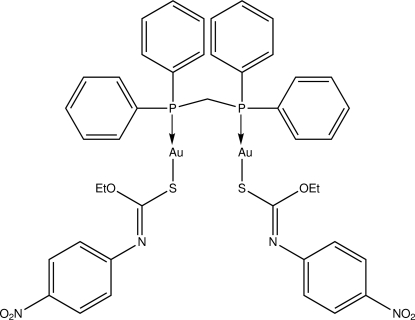


## Experimental

### 

#### Crystal data

[Au_2_(C_9_H_9_N_2_O_3_S)_2_(C_25_H_22_P_2_)]
*M* *_r_* = 1228.83
Monoclinic, 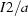

*a* = 24.400 (3) Å
*b* = 16.1419 (16) Å
*c* = 24.594 (2) Å
β = 110.252 (9)°
*V* = 9087.9 (16) Å^3^
*Z* = 8
Mo *K*α radiation
μ = 6.66 mm^−1^
*T* = 223 K
0.31 × 0.13 × 0.05 mm

#### Data collection

Bruker SMART CCD diffractometer
Absorption correction: multi-scan (*SADABS*; Bruker, 2000 ▶) *T*_min_ = 0.445, *T*_max_ = 1
31967 measured reflections
10427 independent reflections
7923 reflections with *I* > 2σ(*I*)
*R*_int_ = 0.053

#### Refinement

*R*[*F* ^2^ > 2σ(*F* ^2^)] = 0.039
*wR*(*F* ^2^) = 0.110
*S* = 1.02
10427 reflections
549 parameters
28 restraints
H-atom parameters constrained
Δρ_max_ = 1.52 e Å^−3^
Δρ_min_ = −1.19 e Å^−3^

### 

Data collection: *SMART* (Bruker, 2000 ▶); cell refinement: *SAINT* (Bruker, 2000 ▶); data reduction: *SAINT* ; program(s) used to solve structure: *PATTY* in *DIRDIF92* (Beurskens *et al.*, 1992 ▶); program(s) used to refine structure: *SHELXL97* (Sheldrick, 2008 ▶); molecular graphics: *ORTEP-3* (Farrugia, 1997 ▶) and *DIAMOND* (Brandenburg, 2006 ▶); software used to prepare material for publication: *publCIF* (Westrip, 2010 ▶).

## Supplementary Material

Crystal structure: contains datablocks global, I. DOI: 10.1107/S1600536810016326/hb5435sup1.cif

Structure factors: contains datablocks I. DOI: 10.1107/S1600536810016326/hb5435Isup2.hkl

Additional supplementary materials:  crystallographic information; 3D view; checkCIF report

## Figures and Tables

**Table 1:** Selected bond lengths (Å)

Au1—P1	2.2582 (15)
Au1—S1	2.3087 (16)
Au2—P2	2.2421 (15)
Au2—S2	2.3012 (16)

**Table 2:** Hydrogen-bond geometry (Å, °) *Cg*1 and *Cg*2 are the centroids of the C2–C7 and C38–C43 rings, respectively.

*D*—H⋯*A*	*D*—H	H⋯*A*	*D*⋯*A*	*D*—H⋯*A*
C41—H41⋯*Cg*1^i^	0.94	2.73	3.576 (8)	151
C17—H17b⋯*Cg*2^ii^	0.98	2.87	3.821 (11)	163

Symmetry codes: (i) 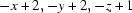; (ii) 
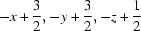
.

**Fig. 1.**
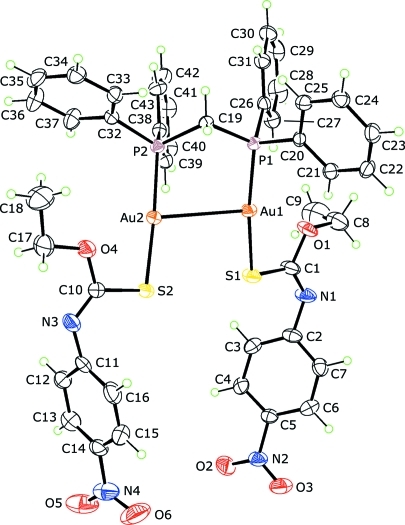
Molecular structure of (I) showing displacement ellipsoids at the 50% probability level. Only the major component of the disordered ethoxy group is shown for reasons of clarity.

**Fig. 2.**
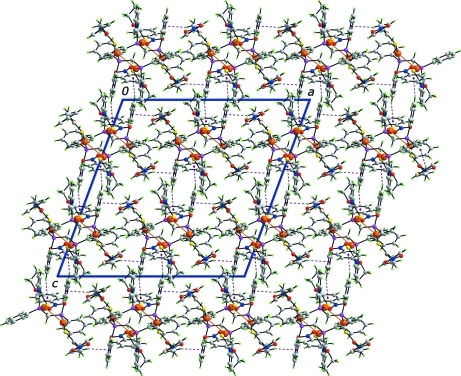
A view in projection down the b axis of the crystal packing in (I). The C–H···π contacts are shown as purple dashed lines.

## supplementary crystallographic information

### Comment

The investigation of dinuclear molecules related to molecules with the general formula R_3_PAu[SC(OR')═ NR''], for R, R' and R'' = alkyl and aryl,have proved useful for crystal engineering studies, in particular in terms of a competition between intra- and inter-molecular aurophilic (Au···Au) interactions, and the influence of these upon luminescence (Ho *et al.*, 2006; Ho & Tiekink, 2007; Kuan *et al.*, 2008). The title compound, (I), is the ethoxy analogue of the previously reported methoxy derivative (Ho *et al.*, 2006).

The nearly linear *SP* coordination geometry observed for each Au atom, Fig. 1, is defined by one P atom of the bidentate bridging diphosphine ligand and the thiolate-S derived from the carbonimidothioate anion, Table 1. Deviations from the ideal linearity [S—Au—P = 172.77 (6) and 173.84 (6) °] is traced to the close intramolecular Au···O contacts [2.968 (11) and 2.963 (4) Å]. Overall, the conformation of the dinuclear molecule is a U-shape which allows for the formation of an intramolecular Au···Au contact of 3.2320 (5) Å which is longer than 3.1589 (4) Å found in the methoxy derivative (Ho *et al.* 2006).

The major feature of the crystal packing is the presence of C–H···π interactions, Table 2 and Fig. 2.

### Experimental

Compound (I) was prepared following the standard literature procedure from the reaction of [Ph_2_PCH_2_PPh_2_](AuCl)_2_ and EtOC(═S)N(H)(C_6_H_4_NO_2_-4) in the presence of NaOH (Hall *et al.*, 1993). Yellow blocks of (I) were obtained by the slow evaporation of a CHCl_3_/hexane (3/1) solution held at room temperature; m.pt. 483 K. Analysis, Found (Calculated): C 41.97 (42.03); H 3.67 (3.28); N 4.09 (4.56); S 4.64 (5.20). IR (KBr, cm^-1^): ν(C–S) 1103 (s), 851 (m); ν(C–N) 1580 (m); ν(C–O) 1144 (s). ^31^P{^1^H} (CDCl_3_) NMR: δ 29.2 p.p.m.

### Refinement

The H atoms were geometrically placed (C—H = 0.94–0.98 Å) and refined as riding with *U*_iso_(H) = 1.2-1.5*U*_eq_(C). The maximum and minimum residual electron density peaks of 1.52 and 1.19 e Å^-3^, respectively, were located 0.90 Å and 1.53 Å from the Au1 and Au2 atoms, respectively. High thermal motion was noted in the O1-ethoxy substituent but only two positions were resolved for each of three atoms. Anisotropic refinement (constrained to be equivalent for paired components of the disorder, and approximately isotropic by the EADP and ISOR commands in SHELXL-97 (Sheldrick, 2008), respectively) and with the O–C and C–C distances restrained to 1.45+0.01 and 1.48±0.01 Å showed the major component of the disorder had a site occupancy factor = 0.679 (16).

### Crystal data

**Table:**

[Au_2_(C_9_H_9_N_2_O_3_S)_2_(C_25_H_22_P_2_)]	*F*(000) = 4751
*M**_r_* = 1228.83	*D*_x_ = 1.796 Mg m^−^^3^
Monoclinic, *I*2/*a*	Mo *K*α radiation, λ = 0.71069 Å
Hall symbol: -I 2ya	Cell parameters from 5597 reflections
*a* = 24.400 (3) Å	θ = 2.5–24.2°
*b* = 16.1419 (16) Å	µ = 6.66 mm^−^^1^
*c* = 24.594 (2) Å	*T* = 223 K
β = 110.252 (9)°	Block, yellow
*V* = 9087.9 (16) Å^3^	0.31 × 0.13 × 0.05 mm
*Z* = 8	

### Data collection

**Table:**

Bruker SMART CCD diffractometer	10427 independent reflections
Radiation source: fine-focus sealed tube	7923 reflections with *I* > 2σ(*I*)
graphite	*R*_int_ = 0.053
ω scans	θ_max_ = 27.5°, θ_min_ = 1.5°
Absorption correction: multi-scan (*SADABS*; Bruker, 2000)	*h* = −27→31
*T*_min_ = 0.445, *T*_max_ = 1	*k* = −20→20
31967 measured reflections	*l* = −31→24

### Refinement

**Table:**

Refinement on *F*^2^	Primary atom site location: structure-invariant direct methods
Least-squares matrix: full	Secondary atom site location: difference Fourier map
*R*[*F*^2^ > 2σ(*F*^2^)] = 0.039	Hydrogen site location: inferred from neighbouring sites
*wR*(*F*^2^) = 0.110	H-atom parameters constrained
*S* = 1.02	*w* = 1/[σ^2^(*F*_o_^2^) + (0.0567*P*)^2^] where *P* = (*F*_o_^2^ + 2*F*_c_^2^)/3
10427 reflections	(Δ/σ)_max_ = 0.001
549 parameters	Δρ_max_ = 1.52 e Å^−^^3^
28 restraints	Δρ_min_ = −1.19 e Å^−^^3^

### Special details

**Table:**

Geometry. All s.u.'s (except the s.u. in the dihedral angle between two l.s. planes) are estimated using the full covariance matrix. The cell s.u.'s are taken into account individually in the estimation of s.u.'s in distances, angles and torsion angles; correlations between s.u.'s in cell parameters are only used when they are defined by crystal symmetry. An approximate (isotropic) treatment of cell s.u.'s is used for estimating s.u.'s involving l.s. planes.
Refinement. Refinement of *F*^2^ against ALL reflections. The weighted *R*-factor wR and goodness of fit *S* are based on *F*^2^, conventional *R*-factors *R* are based on *F*, with *F* set to zero for negative *F*^2^. The threshold expression of *F*^2^ > 2σ(*F*^2^) is used only for calculating *R*-factors(gt) etc. and is not relevant to the choice of reflections for refinement. *R*-factors based on *F*^2^ are statistically about twice as large as those based on *F*, and *R*- factors based on ALL data will be even larger.

### Fractional atomic coordinates and isotropic or equivalent isotropic displacement parameters (Å^2^)

**Table:**

	*x*	*y*	*z*	*U*_iso_*/*U*_eq_	Occ. (<1)
Au1	1.009521 (10)	0.859132 (13)	0.324297 (11)	0.03469 (8)	
Au2	0.879170 (10)	0.815714 (13)	0.237856 (11)	0.03270 (8)	
S1	0.99717 (8)	0.99888 (10)	0.33639 (8)	0.0448 (4)	
S2	0.84758 (7)	0.93438 (9)	0.18429 (8)	0.0408 (4)	
P1	1.03352 (6)	0.72491 (9)	0.31943 (7)	0.0290 (3)	
P2	0.90430 (7)	0.69252 (9)	0.28151 (7)	0.0288 (3)	
O2	0.9091 (3)	1.4064 (3)	0.3450 (3)	0.0745 (18)	
O3	0.9791 (3)	1.4426 (3)	0.3166 (3)	0.0731 (17)	
O4	0.7826 (2)	0.8134 (3)	0.1245 (2)	0.0553 (14)	
O5	0.7025 (4)	1.3205 (4)	0.1153 (3)	0.096 (3)	
O6	0.7716 (4)	1.3265 (4)	0.0808 (4)	0.111 (3)	
N1	1.0713 (3)	1.0927 (3)	0.4211 (3)	0.0595 (18)	
N2	0.9560 (3)	1.3930 (3)	0.3388 (3)	0.0514 (16)	
N3	0.7433 (2)	0.9417 (4)	0.0988 (3)	0.0548 (17)	
N4	0.7390 (4)	1.2877 (4)	0.0992 (3)	0.071 (2)	
C1	1.0559 (3)	1.0213 (4)	0.4003 (3)	0.0517 (19)	
C2	1.0404 (3)	1.1643 (4)	0.3968 (3)	0.0430 (17)	
C3	0.9831 (3)	1.1771 (4)	0.3941 (3)	0.0470 (17)	
H3	0.9633	1.1343	0.4055	0.056*	
C4	0.9546 (3)	1.2513 (4)	0.3751 (3)	0.0421 (16)	
H4	0.9160	1.2596	0.3735	0.051*	
C5	0.9849 (3)	1.3130 (3)	0.3584 (3)	0.0366 (14)	
C6	1.0423 (3)	1.3030 (4)	0.3607 (3)	0.0422 (16)	
H6	1.0622	1.3460	0.3496	0.051*	
C7	1.0689 (3)	1.2284 (4)	0.3796 (3)	0.0458 (16)	
H7	1.1075	1.2202	0.3810	0.055*	
O1	1.0910 (5)	0.9539 (9)	0.4226 (5)	0.053 (3)	0.679 (16)
C8	1.1390 (6)	0.9687 (10)	0.4772 (5)	0.071 (4)	0.679 (16)
H8A	1.1583	1.0211	0.4749	0.085*	0.679 (16)
H8B	1.1679	0.9241	0.4840	0.085*	0.679 (16)
C9	1.1167 (9)	0.9720 (13)	0.5257 (9)	0.129 (7)	0.679 (16)
H9A	1.0805	1.0033	0.5140	0.194*	0.679 (16)
H9B	1.1453	0.9986	0.5587	0.194*	0.679 (16)
H9C	1.1094	0.9161	0.5361	0.194*	0.679 (16)
O1A	1.0752 (12)	0.955 (2)	0.4357 (11)	0.053 (3)	0.321 (16)
C8A	1.1194 (13)	0.962 (3)	0.4932 (13)	0.071 (4)	0.321 (16)
H8C	1.1197	0.9118	0.5154	0.085*	0.321 (16)
H8D	1.1104	1.0092	0.5139	0.085*	0.321 (16)
C9A	1.1774 (18)	0.974 (3)	0.488 (2)	0.129 (7)	0.321 (16)
H9D	1.1897	0.9235	0.4746	0.194*	0.321 (16)
H9E	1.2055	0.9895	0.5254	0.194*	0.321 (16)
H9F	1.1751	1.0184	0.4603	0.194*	0.321 (16)
C10	0.7841 (3)	0.8975 (4)	0.1301 (3)	0.0420 (16)	
C11	0.7461 (3)	1.0285 (4)	0.1030 (3)	0.0500 (19)	
C12	0.7136 (3)	1.0705 (4)	0.1311 (3)	0.0457 (17)	
H12	0.6928	1.0406	0.1504	0.055*	
C13	0.7120 (3)	1.1557 (5)	0.1307 (3)	0.0523 (18)	
H13	0.6905	1.1844	0.1499	0.063*	
C14	0.7421 (3)	1.1983 (4)	0.1020 (3)	0.0501 (19)	
C15	0.7755 (4)	1.1598 (5)	0.0743 (4)	0.064 (2)	
H15	0.7970	1.1910	0.0564	0.076*	
C16	0.7768 (3)	1.0742 (5)	0.0736 (4)	0.060 (2)	
H16	0.7981	1.0465	0.0537	0.072*	
C17	0.7306 (4)	0.7781 (5)	0.0805 (4)	0.073 (3)	
H17A	0.7252	0.8022	0.0425	0.087*	
H17B	0.6957	0.7900	0.0903	0.087*	
C18	0.7394 (5)	0.6876 (5)	0.0793 (5)	0.105 (4)	
H18A	0.7737	0.6764	0.0689	0.157*	
H18B	0.7054	0.6624	0.0508	0.157*	
H18C	0.7450	0.6644	0.1172	0.157*	
C19	0.9736 (2)	0.6572 (3)	0.2764 (3)	0.0286 (12)	
H19A	0.9712	0.6578	0.2358	0.034*	
H19B	0.9814	0.6001	0.2907	0.034*	
C20	1.0897 (2)	0.7147 (4)	0.2871 (3)	0.0292 (12)	
C21	1.1218 (3)	0.7832 (4)	0.2835 (3)	0.0387 (14)	
H21	1.1139	0.8347	0.2970	0.046*	
C22	1.1651 (3)	0.7771 (4)	0.2603 (3)	0.0447 (16)	
H22	1.1870	0.8243	0.2584	0.054*	
C23	1.1770 (3)	0.7024 (4)	0.2398 (3)	0.0461 (17)	
H23	1.2065	0.6991	0.2234	0.055*	
C24	1.1458 (3)	0.6324 (4)	0.2431 (3)	0.0434 (16)	
H24	1.1542	0.5812	0.2296	0.052*	
C25	1.1021 (3)	0.6386 (4)	0.2667 (3)	0.0377 (14)	
H25	1.0805	0.5913	0.2689	0.045*	
C26	1.0615 (3)	0.6760 (4)	0.3902 (3)	0.0346 (14)	
C27	1.0727 (3)	0.7253 (5)	0.4402 (3)	0.0447 (16)	
H27	1.0668	0.7829	0.4369	0.054*	
C28	1.0923 (3)	0.6884 (6)	0.4935 (3)	0.061 (2)	
H28	1.1002	0.7213	0.5269	0.073*	
C29	1.1006 (3)	0.6060 (6)	0.4992 (3)	0.061 (2)	
H29	1.1136	0.5820	0.5363	0.074*	
C30	1.0899 (3)	0.5563 (5)	0.4507 (3)	0.0519 (19)	
H30	1.0957	0.4987	0.4551	0.062*	
C31	1.0707 (3)	0.5910 (4)	0.3953 (3)	0.0413 (15)	
H31	1.0642	0.5578	0.3623	0.050*	
C32	0.8515 (2)	0.6121 (4)	0.2458 (3)	0.0328 (13)	
C33	0.8615 (3)	0.5573 (4)	0.2072 (3)	0.0415 (16)	
H33	0.8973	0.5578	0.2006	0.050*	
C34	0.8178 (3)	0.5010 (4)	0.1779 (3)	0.0481 (17)	
H34	0.8242	0.4634	0.1515	0.058*	
C35	0.7657 (3)	0.4999 (4)	0.1874 (3)	0.055 (2)	
H35	0.7364	0.4623	0.1670	0.066*	
C36	0.7564 (4)	0.5527 (5)	0.2257 (4)	0.062 (2)	
H36	0.7208	0.5513	0.2324	0.075*	
C37	0.7994 (3)	0.6095 (5)	0.2554 (4)	0.0527 (19)	
H37	0.7927	0.6461	0.2822	0.063*	
C38	0.9131 (2)	0.6901 (3)	0.3575 (3)	0.0315 (13)	
C39	0.9128 (3)	0.7625 (4)	0.3875 (3)	0.0413 (15)	
H39	0.9056	0.8133	0.3675	0.050*	
C40	0.9232 (3)	0.7613 (5)	0.4467 (3)	0.0521 (19)	
H40	0.9224	0.8109	0.4665	0.063*	
C41	0.9344 (3)	0.6881 (5)	0.4761 (3)	0.054 (2)	
H41	0.9424	0.6876	0.5163	0.065*	
C42	0.9342 (3)	0.6141 (5)	0.4468 (3)	0.056 (2)	
H42	0.9413	0.5637	0.4672	0.067*	
C43	0.9234 (3)	0.6149 (4)	0.3873 (3)	0.0415 (15)	
H43	0.9232	0.5651	0.3673	0.050*	

### Atomic displacement parameters (Å^2^)

**Table:**

	*U*^11^	*U*^22^	*U*^33^	*U*^12^	*U*^13^	*U*^23^
Au1	0.03798 (14)	0.02598 (12)	0.03917 (15)	0.00068 (9)	0.01218 (11)	−0.00119 (10)
Au2	0.03173 (13)	0.02833 (12)	0.03663 (14)	0.00304 (9)	0.01005 (10)	0.00221 (10)
S1	0.0507 (10)	0.0294 (8)	0.0440 (10)	0.0059 (7)	0.0033 (8)	−0.0017 (7)
S2	0.0376 (8)	0.0290 (7)	0.0470 (10)	0.0019 (6)	0.0034 (7)	0.0043 (7)
P1	0.0299 (7)	0.0259 (7)	0.0310 (8)	−0.0013 (6)	0.0102 (6)	0.0011 (6)
P2	0.0301 (8)	0.0263 (7)	0.0300 (8)	−0.0007 (6)	0.0105 (6)	−0.0013 (6)
O2	0.058 (4)	0.046 (3)	0.119 (6)	0.014 (3)	0.030 (4)	−0.007 (3)
O3	0.085 (4)	0.040 (3)	0.088 (5)	0.002 (3)	0.023 (4)	0.014 (3)
O4	0.047 (3)	0.039 (3)	0.065 (4)	0.001 (2)	0.000 (3)	−0.002 (2)
O5	0.152 (7)	0.048 (4)	0.078 (5)	0.023 (4)	0.028 (5)	0.001 (3)
O6	0.141 (7)	0.057 (4)	0.117 (7)	−0.035 (4)	0.023 (6)	0.013 (4)
N1	0.064 (4)	0.028 (3)	0.064 (4)	0.005 (3)	−0.007 (3)	−0.005 (3)
N2	0.057 (4)	0.030 (3)	0.057 (4)	0.001 (3)	0.007 (3)	−0.001 (3)
N3	0.039 (3)	0.046 (3)	0.062 (4)	0.005 (3)	−0.005 (3)	0.001 (3)
N4	0.098 (6)	0.043 (4)	0.049 (4)	−0.004 (4)	−0.006 (4)	−0.001 (3)
C1	0.053 (4)	0.032 (3)	0.057 (5)	0.012 (3)	0.002 (4)	0.003 (3)
C2	0.057 (4)	0.029 (3)	0.033 (4)	0.007 (3)	0.003 (3)	−0.005 (3)
C3	0.057 (4)	0.034 (3)	0.053 (5)	−0.008 (3)	0.024 (4)	−0.004 (3)
C4	0.041 (4)	0.036 (3)	0.045 (4)	−0.008 (3)	0.010 (3)	−0.014 (3)
C5	0.045 (4)	0.028 (3)	0.035 (4)	0.003 (3)	0.011 (3)	−0.005 (3)
C6	0.063 (4)	0.034 (3)	0.038 (4)	−0.006 (3)	0.028 (3)	−0.005 (3)
C7	0.040 (4)	0.048 (4)	0.051 (4)	0.002 (3)	0.018 (3)	−0.007 (3)
O1	0.052 (7)	0.036 (3)	0.055 (6)	0.020 (5)	−0.003 (4)	−0.005 (5)
C8	0.070 (7)	0.061 (5)	0.071 (6)	0.016 (5)	0.008 (5)	0.005 (5)
C9	0.137 (9)	0.116 (8)	0.122 (9)	0.011 (6)	0.028 (6)	0.001 (6)
O1A	0.052 (7)	0.036 (3)	0.055 (6)	0.020 (5)	−0.003 (4)	−0.005 (5)
C8A	0.070 (7)	0.061 (5)	0.071 (6)	0.016 (5)	0.008 (5)	0.005 (5)
C9A	0.137 (9)	0.116 (8)	0.122 (9)	0.011 (6)	0.028 (6)	0.001 (6)
C10	0.038 (3)	0.034 (3)	0.049 (4)	0.000 (3)	0.009 (3)	0.005 (3)
C11	0.030 (3)	0.045 (4)	0.061 (5)	0.009 (3)	−0.003 (3)	0.013 (4)
C12	0.044 (4)	0.048 (4)	0.039 (4)	−0.001 (3)	0.008 (3)	0.004 (3)
C13	0.054 (5)	0.058 (4)	0.041 (4)	0.006 (4)	0.012 (4)	−0.006 (4)
C14	0.047 (4)	0.042 (4)	0.045 (4)	0.002 (3)	−0.004 (3)	0.006 (3)
C15	0.047 (4)	0.064 (5)	0.075 (6)	−0.011 (4)	0.014 (4)	0.014 (5)
C16	0.039 (4)	0.066 (5)	0.076 (6)	0.011 (4)	0.021 (4)	−0.002 (4)
C17	0.056 (5)	0.061 (5)	0.077 (6)	−0.006 (4)	−0.008 (5)	−0.010 (5)
C18	0.108 (9)	0.053 (5)	0.126 (11)	−0.011 (5)	0.005 (8)	−0.025 (6)
C19	0.031 (3)	0.026 (3)	0.029 (3)	−0.001 (2)	0.011 (2)	−0.001 (2)
C20	0.022 (3)	0.035 (3)	0.029 (3)	−0.002 (2)	0.007 (2)	0.005 (3)
C21	0.035 (3)	0.036 (3)	0.039 (4)	−0.002 (3)	0.005 (3)	0.001 (3)
C22	0.036 (3)	0.048 (4)	0.048 (4)	−0.010 (3)	0.013 (3)	0.010 (3)
C23	0.035 (4)	0.057 (4)	0.049 (4)	0.001 (3)	0.019 (3)	0.008 (3)
C24	0.044 (4)	0.043 (4)	0.048 (4)	0.007 (3)	0.023 (3)	0.006 (3)
C25	0.041 (3)	0.031 (3)	0.043 (4)	0.002 (3)	0.016 (3)	0.000 (3)
C26	0.025 (3)	0.044 (3)	0.032 (3)	−0.001 (2)	0.005 (3)	0.004 (3)
C27	0.042 (4)	0.052 (4)	0.037 (4)	0.000 (3)	0.009 (3)	−0.001 (3)
C28	0.056 (5)	0.092 (7)	0.026 (4)	0.001 (4)	0.003 (3)	−0.001 (4)
C29	0.044 (4)	0.097 (7)	0.032 (4)	0.002 (4)	−0.001 (3)	0.027 (4)
C30	0.044 (4)	0.054 (4)	0.054 (5)	0.007 (3)	0.012 (4)	0.026 (4)
C31	0.039 (4)	0.041 (3)	0.043 (4)	0.004 (3)	0.015 (3)	0.008 (3)
C32	0.029 (3)	0.032 (3)	0.037 (3)	0.001 (2)	0.010 (3)	0.005 (3)
C33	0.034 (3)	0.036 (3)	0.051 (4)	−0.001 (3)	0.010 (3)	−0.007 (3)
C34	0.051 (4)	0.036 (3)	0.049 (4)	−0.003 (3)	0.006 (3)	−0.010 (3)
C35	0.058 (5)	0.041 (4)	0.052 (5)	−0.021 (3)	0.001 (4)	−0.002 (4)
C36	0.052 (5)	0.069 (5)	0.070 (6)	−0.026 (4)	0.026 (4)	−0.007 (5)
C37	0.049 (4)	0.057 (4)	0.059 (5)	−0.012 (4)	0.028 (4)	−0.006 (4)
C38	0.029 (3)	0.034 (3)	0.033 (3)	−0.002 (2)	0.011 (3)	−0.003 (3)
C39	0.040 (4)	0.043 (4)	0.040 (4)	0.002 (3)	0.013 (3)	−0.003 (3)
C40	0.055 (4)	0.058 (5)	0.044 (4)	−0.003 (4)	0.019 (4)	−0.018 (4)
C41	0.054 (5)	0.076 (6)	0.033 (4)	−0.005 (4)	0.015 (3)	−0.003 (4)
C42	0.066 (5)	0.061 (5)	0.042 (4)	−0.008 (4)	0.021 (4)	0.013 (4)
C43	0.054 (4)	0.033 (3)	0.044 (4)	−0.001 (3)	0.025 (3)	0.002 (3)

### Geometric parameters (Å, °)

**Table:**

Au1—P1	2.2582 (15)	C14—C15	1.378 (12)
Au1—S1	2.3087 (16)	C15—C16	1.381 (11)
Au2—P2	2.2421 (15)	C15—H15	0.9400
Au2—S2	2.3012 (16)	C16—H16	0.9400
Au1—Au2	3.2320 (5)	C17—C18	1.479 (11)
S1—C1	1.759 (8)	C17—H17A	0.9800
S2—C10	1.761 (7)	C17—H17B	0.9800
P1—C20	1.815 (6)	C18—H18A	0.9700
P1—C26	1.816 (6)	C18—H18B	0.9700
P1—C19	1.839 (6)	C18—H18C	0.9700
P2—C38	1.808 (6)	C19—H19A	0.9800
P2—C32	1.826 (6)	C19—H19B	0.9800
P2—C19	1.831 (6)	C20—C21	1.377 (8)
O2—N2	1.224 (8)	C20—C25	1.399 (8)
O3—N2	1.213 (8)	C21—C22	1.366 (9)
O4—C10	1.364 (7)	C21—H21	0.9400
O4—C17	1.468 (9)	C22—C23	1.376 (10)
O5—N4	1.215 (11)	C22—H22	0.9400
O6—N4	1.215 (11)	C23—C24	1.381 (9)
N1—C1	1.264 (9)	C23—H23	0.9400
N1—C2	1.397 (8)	C24—C25	1.383 (9)
N2—C5	1.470 (8)	C24—H24	0.9400
N3—C10	1.249 (8)	C25—H25	0.9400
N3—C11	1.405 (9)	C26—C31	1.389 (9)
N4—C14	1.446 (9)	C26—C27	1.410 (9)
C1—O1A	1.35 (4)	C27—C28	1.368 (10)
C1—O1	1.377 (14)	C27—H27	0.9400
C2—C3	1.391 (10)	C28—C29	1.346 (12)
C2—C7	1.392 (10)	C28—H28	0.9400
C3—C4	1.382 (9)	C29—C30	1.387 (11)
C3—H3	0.9400	C29—H29	0.9400
C4—C5	1.384 (9)	C30—C31	1.395 (9)
C4—H4	0.9400	C30—H30	0.9400
C5—C6	1.391 (9)	C31—H31	0.9400
C6—C7	1.371 (9)	C32—C37	1.371 (9)
C6—H6	0.9400	C32—C33	1.381 (9)
C7—H7	0.9400	C33—C34	1.396 (9)
O1—C8	1.465 (9)	C33—H33	0.9400
C8—C9	1.473 (10)	C34—C35	1.368 (10)
C8—H8A	0.9800	C34—H34	0.9400
C8—H8B	0.9800	C35—C36	1.347 (11)
C9—H9A	0.9700	C35—H35	0.9400
C9—H9B	0.9700	C36—C37	1.394 (10)
C9—H9C	0.9700	C36—H36	0.9400
O1A—C8A	1.455 (10)	C37—H37	0.9400
C8A—C9A	1.478 (10)	C38—C39	1.383 (9)
C8A—H8C	0.9800	C38—C43	1.394 (9)
C8A—H8D	0.9800	C39—C40	1.390 (10)
C9A—H9D	0.9700	C39—H39	0.9400
C9A—H9E	0.9700	C40—C41	1.362 (11)
C9A—H9F	0.9700	C40—H40	0.9400
C11—C12	1.395 (10)	C41—C42	1.393 (11)
C11—C16	1.416 (11)	C41—H41	0.9400
C12—C13	1.376 (10)	C42—C43	1.396 (10)
C12—H12	0.9400	C42—H42	0.9400
C13—C14	1.365 (11)	C43—H43	0.9400
C13—H13	0.9400		
			
P1—Au1—S1	172.77 (6)	C15—C16—H16	120.1
P1—Au1—Au2	88.36 (4)	C11—C16—H16	120.1
S1—Au1—Au2	98.86 (4)	O4—C17—C18	107.7 (7)
P2—Au2—S2	173.84 (6)	O4—C17—H17A	110.2
P2—Au2—Au1	80.42 (4)	C18—C17—H17A	110.2
S2—Au2—Au1	104.74 (4)	O4—C17—H17B	110.2
C1—S1—Au1	102.4 (2)	C18—C17—H17B	110.2
C10—S2—Au2	100.7 (2)	H17A—C17—H17B	108.5
C20—P1—C26	106.9 (3)	C17—C18—H18A	109.5
C20—P1—C19	105.3 (3)	C17—C18—H18B	109.5
C26—P1—C19	104.7 (3)	H18A—C18—H18B	109.5
C20—P1—Au1	111.07 (19)	C17—C18—H18C	109.5
C26—P1—Au1	112.6 (2)	H18A—C18—H18C	109.5
C19—P1—Au1	115.60 (19)	H18B—C18—H18C	109.5
C38—P2—C32	107.0 (3)	P2—C19—P1	110.1 (3)
C38—P2—C19	106.5 (3)	P2—C19—H19A	109.6
C32—P2—C19	105.1 (3)	P1—C19—H19A	109.6
C38—P2—Au2	115.44 (19)	P2—C19—H19B	109.6
C32—P2—Au2	111.7 (2)	P1—C19—H19B	109.6
C19—P2—Au2	110.46 (19)	H19A—C19—H19B	108.2
C10—O4—C17	116.5 (5)	C21—C20—C25	118.9 (6)
C1—N1—C2	122.7 (6)	C21—C20—P1	119.3 (5)
O3—N2—O2	122.6 (6)	C25—C20—P1	121.8 (4)
O3—N2—C5	118.8 (6)	C22—C21—C20	120.6 (6)
O2—N2—C5	118.6 (6)	C22—C21—H21	119.7
C10—N3—C11	121.0 (6)	C20—C21—H21	119.7
O6—N4—O5	122.9 (8)	C21—C22—C23	120.5 (6)
O6—N4—C14	120.2 (10)	C21—C22—H22	119.7
O5—N4—C14	116.9 (9)	C23—C22—H22	119.7
N1—C1—O1A	117.8 (14)	C22—C23—C24	120.3 (6)
N1—C1—O1	120.2 (8)	C22—C23—H23	119.9
O1A—C1—O1	24.6 (12)	C24—C23—H23	119.9
N1—C1—S1	125.9 (5)	C23—C24—C25	119.2 (6)
O1A—C1—S1	114.1 (14)	C23—C24—H24	120.4
O1—C1—S1	113.3 (7)	C25—C24—H24	120.4
C3—C2—C7	118.3 (6)	C24—C25—C20	120.5 (6)
C3—C2—N1	122.0 (7)	C24—C25—H25	119.8
C7—C2—N1	119.4 (7)	C20—C25—H25	119.8
C4—C3—C2	121.6 (6)	C31—C26—C27	120.2 (6)
C4—C3—H3	119.2	C31—C26—P1	120.8 (5)
C2—C3—H3	119.2	C27—C26—P1	119.0 (5)
C3—C4—C5	117.8 (6)	C28—C27—C26	119.2 (7)
C3—C4—H4	121.1	C28—C27—H27	120.4
C5—C4—H4	121.1	C26—C27—H27	120.4
C4—C5—C6	122.5 (6)	C29—C28—C27	121.4 (8)
C4—C5—N2	118.9 (6)	C29—C28—H28	119.3
C6—C5—N2	118.6 (6)	C27—C28—H28	119.3
C7—C6—C5	117.9 (6)	C28—C29—C30	120.4 (7)
C7—C6—H6	121.1	C28—C29—H29	119.8
C5—C6—H6	121.1	C30—C29—H29	119.8
C6—C7—C2	121.9 (6)	C29—C30—C31	120.4 (7)
C6—C7—H7	119.1	C29—C30—H30	119.8
C2—C7—H7	119.1	C31—C30—H30	119.8
C1—O1—C8	115.3 (12)	C26—C31—C30	118.3 (7)
O1—C8—C9	110.1 (17)	C26—C31—H31	120.8
O1—C8—H8A	109.6	C30—C31—H31	120.8
C9—C8—H8A	109.6	C37—C32—C33	119.6 (6)
O1—C8—H8B	109.6	C37—C32—P2	118.7 (5)
C9—C8—H8B	109.6	C33—C32—P2	121.5 (5)
H8A—C8—H8B	108.1	C32—C33—C34	119.0 (6)
C1—O1A—C8A	123 (3)	C32—C33—H33	120.5
O1A—C8A—C9A	110 (4)	C34—C33—H33	120.5
O1A—C8A—H8C	109.7	C35—C34—C33	120.7 (7)
C9A—C8A—H8C	109.7	C35—C34—H34	119.7
O1A—C8A—H8D	109.7	C33—C34—H34	119.7
C9A—C8A—H8D	109.7	C36—C35—C34	120.1 (7)
H8C—C8A—H8D	108.2	C36—C35—H35	119.9
C8A—C9A—H9D	109.5	C34—C35—H35	119.9
C8A—C9A—H9E	109.5	C35—C36—C37	120.3 (7)
H9D—C9A—H9E	109.5	C35—C36—H36	119.8
C8A—C9A—H9F	109.5	C37—C36—H36	119.8
H9D—C9A—H9F	109.5	C32—C37—C36	120.2 (7)
H9E—C9A—H9F	109.5	C32—C37—H37	119.9
N3—C10—O4	121.2 (6)	C36—C37—H37	119.9
N3—C10—S2	125.3 (5)	C39—C38—C43	119.4 (6)
O4—C10—S2	113.5 (5)	C39—C38—P2	120.8 (5)
C12—C11—N3	120.0 (7)	C43—C38—P2	119.8 (5)
C12—C11—C16	119.5 (7)	C38—C39—C40	120.9 (7)
N3—C11—C16	120.2 (7)	C38—C39—H39	119.6
C13—C12—C11	120.1 (7)	C40—C39—H39	119.6
C13—C12—H12	120.0	C41—C40—C39	119.9 (7)
C11—C12—H12	120.0	C41—C40—H40	120.0
C14—C13—C12	119.2 (7)	C39—C40—H40	120.0
C14—C13—H13	120.4	C40—C41—C42	120.4 (7)
C12—C13—H13	120.4	C40—C41—H41	119.8
C13—C14—C15	123.0 (7)	C42—C41—H41	119.8
C13—C14—N4	119.9 (8)	C41—C42—C43	120.0 (7)
C15—C14—N4	117.2 (8)	C41—C42—H42	120.0
C14—C15—C16	118.5 (8)	C43—C42—H42	120.0
C14—C15—H15	120.8	C38—C43—C42	119.4 (7)
C16—C15—H15	120.8	C38—C43—H43	120.3
C15—C16—C11	119.8 (8)	C42—C43—H43	120.3
			
P1—Au1—Au2—P2	28.86 (6)	N4—C14—C15—C16	−176.7 (7)
S1—Au1—Au2—P2	−151.57 (7)	C14—C15—C16—C11	−2.6 (12)
P1—Au1—Au2—S2	−147.71 (6)	C12—C11—C16—C15	1.6 (11)
S1—Au1—Au2—S2	31.86 (7)	N3—C11—C16—C15	175.0 (7)
P1—Au1—S1—C1	−22.7 (6)	C10—O4—C17—C18	176.7 (8)
Au2—Au1—S1—C1	160.7 (3)	C38—P2—C19—P1	−58.8 (4)
P2—Au2—S2—C10	22.0 (7)	C32—P2—C19—P1	−172.1 (3)
Au1—Au2—S2—C10	168.6 (2)	Au2—P2—C19—P1	67.2 (3)
S1—Au1—P1—C20	−59.6 (6)	C20—P1—C19—P2	−155.5 (3)
Au2—Au1—P1—C20	117.0 (2)	C26—P1—C19—P2	92.0 (3)
S1—Au1—P1—C26	60.2 (6)	Au1—P1—C19—P2	−32.5 (4)
Au2—Au1—P1—C26	−123.2 (2)	C26—P1—C20—C21	−106.5 (5)
S1—Au1—P1—C19	−180 (100)	C19—P1—C20—C21	142.6 (5)
Au2—Au1—P1—C19	−2.9 (2)	Au1—P1—C20—C21	16.7 (5)
S2—Au2—P2—C38	−145.2 (6)	C26—P1—C20—C25	72.4 (5)
Au1—Au2—P2—C38	67.5 (2)	C19—P1—C20—C25	−38.6 (6)
S2—Au2—P2—C32	−22.6 (6)	Au1—P1—C20—C25	−164.4 (4)
Au1—Au2—P2—C32	−170.0 (2)	C25—C20—C21—C22	0.0 (9)
S2—Au2—P2—C19	94.0 (6)	P1—C20—C21—C22	178.8 (5)
Au1—Au2—P2—C19	−53.4 (2)	C20—C21—C22—C23	0.5 (10)
C2—N1—C1—O1A	−158.0 (15)	C21—C22—C23—C24	−0.9 (11)
C2—N1—C1—O1	173.9 (10)	C22—C23—C24—C25	0.8 (11)
C2—N1—C1—S1	4.2 (13)	C23—C24—C25—C20	−0.3 (10)
Au1—S1—C1—N1	171.7 (8)	C21—C20—C25—C24	−0.1 (10)
Au1—S1—C1—O1A	−25.6 (14)	P1—C20—C25—C24	−178.9 (5)
Au1—S1—C1—O1	1.4 (9)	C20—P1—C26—C31	−66.9 (6)
C1—N1—C2—C3	63.0 (12)	C19—P1—C26—C31	44.5 (6)
C1—N1—C2—C7	−123.0 (9)	Au1—P1—C26—C31	170.8 (4)
C7—C2—C3—C4	−0.2 (11)	C20—P1—C26—C27	114.3 (5)
N1—C2—C3—C4	173.8 (7)	C19—P1—C26—C27	−134.4 (5)
C2—C3—C4—C5	0.3 (11)	Au1—P1—C26—C27	−8.0 (6)
C3—C4—C5—C6	−0.6 (10)	C31—C26—C27—C28	−0.5 (10)
C3—C4—C5—N2	−179.1 (6)	P1—C26—C27—C28	178.3 (6)
O3—N2—C5—C4	−169.8 (6)	C26—C27—C28—C29	−0.6 (12)
O2—N2—C5—C4	9.5 (10)	C27—C28—C29—C30	0.8 (12)
O3—N2—C5—C6	11.7 (10)	C28—C29—C30—C31	0.1 (11)
O2—N2—C5—C6	−169.1 (6)	C27—C26—C31—C30	1.4 (9)
C4—C5—C6—C7	0.9 (10)	P1—C26—C31—C30	−177.4 (5)
N2—C5—C6—C7	179.4 (6)	C29—C30—C31—C26	−1.3 (10)
C5—C6—C7—C2	−0.8 (10)	C38—P2—C32—C37	50.4 (6)
C3—C2—C7—C6	0.5 (10)	C19—P2—C32—C37	163.3 (6)
N1—C2—C7—C6	−173.7 (7)	Au2—P2—C32—C37	−76.9 (6)
N1—C1—O1—C8	11.6 (19)	C38—P2—C32—C33	−133.3 (5)
O1A—C1—O1—C8	−80 (4)	C19—P2—C32—C33	−20.4 (6)
S1—C1—O1—C8	−177.5 (11)	Au2—P2—C32—C33	99.4 (5)
C1—O1—C8—C9	76.0 (19)	C37—C32—C33—C34	0.9 (10)
N1—C1—O1A—C8A	−10 (4)	P2—C32—C33—C34	−175.3 (5)
O1—C1—O1A—C8A	93 (5)	C32—C33—C34—C35	0.0 (11)
S1—C1—O1A—C8A	−174 (2)	C33—C34—C35—C36	−0.9 (12)
C1—O1A—C8A—C9A	−74 (4)	C34—C35—C36—C37	0.9 (13)
C11—N3—C10—O4	−177.3 (7)	C33—C32—C37—C36	−1.0 (11)
C11—N3—C10—S2	2.8 (12)	P2—C32—C37—C36	175.4 (6)
C17—O4—C10—N3	−0.6 (11)	C35—C36—C37—C32	0.1 (13)
C17—O4—C10—S2	179.3 (6)	C32—P2—C38—C39	−134.5 (5)
Au2—S2—C10—N3	161.0 (7)	C19—P2—C38—C39	113.6 (5)
Au2—S2—C10—O4	−18.9 (6)	Au2—P2—C38—C39	−9.4 (6)
C10—N3—C11—C12	−104.3 (9)	C32—P2—C38—C43	49.2 (6)
C10—N3—C11—C16	82.4 (10)	C19—P2—C38—C43	−62.8 (5)
N3—C11—C12—C13	−174.0 (6)	Au2—P2—C38—C43	174.2 (4)
C16—C11—C12—C13	−0.6 (11)	C43—C38—C39—C40	0.4 (10)
C11—C12—C13—C14	0.7 (11)	P2—C38—C39—C40	−176.0 (5)
C12—C13—C14—C15	−1.8 (12)	C38—C39—C40—C41	0.8 (11)
C12—C13—C14—N4	177.7 (7)	C39—C40—C41—C42	−1.6 (12)
O6—N4—C14—C13	169.9 (8)	C40—C41—C42—C43	1.1 (12)
O5—N4—C14—C13	−11.0 (11)	C39—C38—C43—C42	−0.9 (10)
O6—N4—C14—C15	−10.6 (11)	P2—C38—C43—C42	175.5 (5)
O5—N4—C14—C15	168.5 (8)	C41—C42—C43—C38	0.1 (11)
C13—C14—C15—C16	2.7 (12)		

### Hydrogen-bond geometry (Å, °)

**Table:**

Cg1 and Cg2 are the centroids of the C2–C7 and C38–C43 rings, respectively.

**Table:**

*D*—H···*A*	*D*—H	H···*A*	*D*···*A*	*D*—H···*A*
C41—H41···Cg1^i^	0.94	2.73	3.576 (8)	151
C17—H17b···Cg2^ii^	0.98	2.87	3.821 (11)	163

Symmetry codes: (i) −*x*+2, −*y*+2, −*z*+1; (ii) −*x*+3/2, −*y*+3/2, −*z*+1/2.
